# Combinational Use of Antiplatelet Medication Sarpogrelate with Therapeutic Drug Rosuvastatin in Treating High-Cholesterol Diet-Induced Chronic Kidney Disease in ApoE-Deficient Mice

**DOI:** 10.1155/2020/1809326

**Published:** 2020-09-26

**Authors:** Jingyi Xu, Zuowei Pei, Meng Yu, Xiang Li, Lu Wang, Yichen Lin, Xinyan Chen, Xiaodan Liu

**Affiliations:** Affiliated Zhongshan Hospital of Dalian University, Dalian, China

## Abstract

A number of metabolic disorders, including hyperlipidemia, potentially cause chronic kidney disease (CKD), one of their major chronic complications and comorbidities. Rosuvastatin is one of the widely used antiatherogenic drugs among hyperlipidemic patients. Meanwhile, sarpogrelate is not only a 5-hydroxytryptamine receptor antagonist but also an antiplatelet agent, inhibiting platelet-stimulated blood coagulation and improving peripheral circulation. In this study, a combination of sarpogrelate and/or rosuvastatin was used on CKD mice induced by a high-fat diet for 8 weeks. The mice were tested for pathological changes using histological evaluation. Tremendous alterations were found, including a remarked increase in total cholesterol and low-density lipoprotein cholesterol levels, glomerular endothelial proliferation, and mesangial expansion. Also, tubular damage and extracellular matrix accumulation occurred, namely, a marked increase in the macula densa, scattered and apoptotic loss of the apical brush border with vacuolated basophilic cytoplasm and heavily stained nuclei, and expanded Bowman's space, which were at least partially ameliorated by sarpogrelate and/or rosuvastatin treatment. The analysis of expression profiles at both the RNA and protein levels, using real-time quantitative polymerase chain reaction and Western blot analysis, indicated that LDL-R/CD68/LOX-1-positive monocyte/macrophage-mediated enhanced proinflammatory activation, including the significant upregulation of tumor necrosis factor-*α* and interleukin-6, was actually attenuated by sarpogrelate and/or rosuvastatin treatment. The findings indicated that sarpogrelate and/or rosuvastatin treatment potentially ameliorates CKD progression in patients with the aforementioned comorbid metabolic disorders.

## 1. Introduction

To date, the complex cellular and molecular nature of chronic kidney disease (CKD) has led to countless failures in clinical trials. Among the causative diseases of CKD, diabetes, hyperlipidemia, hypertension, and obesity have shown strong correlations with CKD [1–6]. Due to the diet and lifestyle in developed countries, most dyslipidemias turn out to be hyperlipidemias, that is, increased levels of apolipoproteins and low-density lipoprotein (LDL) cholesterol and decreased levels of high-density lipoprotein (HDL) cholesterol [1–6]. For instance, the direct binding of apolipoproteins such as ApoE to ATP-binding cassette, subfamily A, member 1 (ABCA1) is essential to ABCA1-mediated cholesterol efflux [7]. Likewise, statins including rosuvastatin [1] or pravastatin [8], the inhibitors of 3-hydroxy-methylglutaryl coenzyme A reductase, are well known for maintaining normal levels of LDL. Obviously, selective alteration in the expression levels of ABCA1 and/or ApoE leads to increased susceptibility to atherosclerosis [9–17].

Atherosclerosis, a major causative factor of cardiovascular diseases, is a chronic inflammatory vascular disease involving an increase in the thickness of arterial walls accompanied by the accumulation of LDL-laden macrophages (foam cells), hyperplasia of smooth muscle cells, and fibrosis, resulting in atheromatous plaques and focus points inside the arterial intimae [9–17]. Specifically, endothelial retention of LDL and its oxidative derivatives (OxLDLs) is the beginning [18], followed by the activation and infiltration of monocytes, B cells, and T cells through toll-like receptor- or scavenger receptor- (SR-) mediated signaling pathways [19, 20], leading to immune responses such as macrophage activation, release of proinflammatory cytokines, production of reactive oxygen species, and lipid accumulation [19, 20].

Meanwhile, OxLDL acts through interactions with LDL-R, or SRs such as CD36 and CD68, or lectin-like oxidized low-density lipoprotein receptor-1 (LOX-1), which is potentially upregulated by proinflammatory or proatherogenic stimuli including tumor necrosis factor-*α* (TNF-*α*), interleukin-6 (IL-6), and interferon-*γ* (IFN-*γ*) [8, 18–20]. Widely expressed LOX-1, along with SRs and LDL-R, were observed in endothelial cells, smooth muscle cells, and even macrophages, indicating the active roles played by LOX-1 in endothelial activation and macrophage activation (foam cell formation) [8, 18–20]. Moreover, a differential expression of CD36, CD68, and other SRs mediates not only the cell-type-specific accumulation and/or degradation of OxLDL but also the inflammatory milieu, revealing its modulation of disease progression [8, 18–20].

Eventually, unstable plaques, which are progressively formed and continuously accumulated, are prone to rupture, resulting in thromboembolism and ischemia [20]. Thus, thrombocyte aggregation and the fibrinolytic/coagulation system are critical during atherosclerosis progression. Sarpogrelate is widely used to prevent arterial thrombosis because it is not only a serotonin receptor antagonist but also an antiplatelet agent [21].

Therefore, this clinical investigation was performed to explore the combinational use of antiplatelet medication such as sarpogrelate with a therapeutic drug such as rosuvastatin in treating CKD caused by atherosclerosis under different conditions [8].

## 2. Materials and Methods

### 2.1. Animal Model

ApoE^−/−^ mice were purchased from Vital River Lab Animal Technology Inc. (Beijing, China). All mice were housed in a room with a 12 : 12 h light-dark cycle with a temperature maintained at 24°C. The 8-week-old male mice were randomly divided into the following four groups: ApoE^−/−^ mice fed a normal diet (*n* = 6); ApoE^−/−^ mice fed a high-cholesterol diet (*n* = 6); ApoE^−/−^ mice fed rosuvastatin calcium [40mg/(kg·d) [22–24]; Mitsubishi Tanabe Pharma, Osaka, Japan]+a high-cholesterol diet (*n* = 6); and ApoE^−/−^ mice fed rosuvastatin calcium [40 mg/(kg·d); Mitsubishi Tanabe Pharma]+sarpogrelate [50 mg/(kg·d); Mitsubishi Tanabe Pharma] [21, 25, 26]+a high-cholesterol diet (*n* = 6). The high-cholesterol diet contained 1.5% (*w*/*w*) cholesterol and 15% (*w*/*w*) fat. The experimental diet was purchased from the Shanghai SLAC Laboratory Animal Inc. (Shanghai, China). The mice in each group were fed their own diet for 8 weeks. After the sacrifice, the kidney weight and the total body weight of the mice from each group were measured and recorded. Blood samples were obtained from their inferior vena cava, collected in serum tubes, and stored at -80°C until use. Coronal sections of the kidneys were fixed in 10% formalin and embedded in paraffin for histological evaluation. The remainder of the kidney was snap-frozen in liquid nitrogen for further transcriptional analysis at either mRNA or protein level. All animal experiments performed in this study were in accordance with the guidelines for the Care and Use of Laboratory Animals and officially approved by the Institutional Animal Care and Use Committee in Zhongshan Affiliated Hospital of Dalian University.

### 2.2. Biochemical Measurements

Serum was obtained and stored at −80°C. TC, TG, high-density lipoprotein (HDL) cholesterol, and LDL-c were measured with an autoanalyzer system (Hitachi 7020, Tokyo, Japan).

### 2.3. Morphologic and Immunohistochemical Analysis

Kidney samples were collected and fixed in 4% paraformaldehyde. Samples embedded in paraffin were cut into slices using a microtome (Leica RM2235 or Leica CM1850UV, Solms, Germany). The slices were then mounted onto glass slides, and immunohistochemistry was performed using the Histone Simple Stain Kit (Nichirei, Tokyo, Japan) according to the manufacturer's protocol. Briefly, paraffin-embedded sections were deparaffinized with xylene and then rehydrated with serially diluted water-ethanol solution. The sections were treated with 3% H_2_O_2_ in methanol to inactivate endogenous peroxidases for 15 min and incubated with primary antibodies for CD68 (rabbit anti-CD68 antibody, 1 : 500; Abcam, UK) or LOX-1 (rabbit anti-LOX-1 antibody, 1 : 250; Abcam) at room temperature for 1 h. All sections were observed with ×40 objective lenses under an upright microscope (Olympus, Tokyo, Japan).

### 2.4. RNA Isolation and Real-Time RT-PCR

Total RNA was isolated from the renal cortex using the Isogen Kit (Nippon Gene, Tokyo, Japan) according to the manufacturer's protocol. Complementary DNA (cDNA) was synthesized using the SuperScript VILO cDNA Synthesis Kit (Life Technologies, CA, USA) according to the manufacturer's protocol. Gene expression was quantitatively analyzed by real-time RT-PCR using fluorescent dye SYBR Green I (Roche LightCycler, Basel/Kaiseraugst, Switzerland). The cDNA of each target gene was amplified, quantitated, and normalized to the amount of *β*-actin gene at the transcriptional level. In addition, the primer sequences used in this study are all listed in Table 1.

### 2.5. Western Blot Analysis in Kidney Tissue

Proteins were extracted from renal cortical tissues using RIPA lysis buffer (Beyotime P0013B, Shanghai, China). Samples were electrophoresed on 10% gel using sodium dodecyl sulfate-polyacrylamide gel electrophoresis, and proteins were then transferred to a polyvinylidene fluoride membrane (Immobilon, Millipore, MA, USA). The membranes were blocked in Tris-buffered saline with 0.1% Tween-20 (TBST) containing 5% skimmed milk, incubated in primary antibody diluents (P0023A; Beyotime), and gently shaken overnight at 4°C. Primary antibodies against LOX-1 (rabbit anti-LOX-1 antibody, 1 : 500; Abcam) and anti-*β*-actin (1 : 1000; Cell Signaling Technology) were added. Then, the membranes were incubated with a secondary antibody (anti-rabbit immunoglobulin G, 1 : 1000; Cell Signaling Technology) for 1 h. All these experiments were repeated independently three times. The expression levels of these proteins were normalized to the amount of *β*-actin proteins to minimize existing differences. The signal intensities were quantified using NIH ImageJ software.

### 2.6. Statistical Analysis

All data were presented as the mean ± standard error of the mean (SEM). The statistical analysis was performed using SPSS software version 23.0 (SPSS Inc., IL, USA). Intergroup variation was determined using one-way analysis of variance followed by Tukey's test. The minimal level of statistical significance was *P* < 0.05.

## 3. Results

### 3.1. Amelioration of Pathological Features in the Renal Cortex in ApoE-Deficient Mice Subjected to a High-Fat Diet Using Rosuvastatin and Sarpogrelate

**Figure 1:**
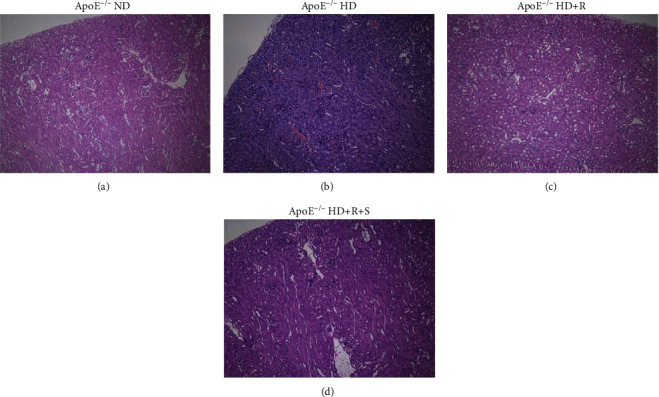
Morphological assessment of kidney tissue under each condition using H&E staining. Using conventional H&E staining, coronal sections of the kidney tissues were used for the histological evaluation of pathological changes in mice randomly divided into four groups: ApoE^−/−^ mice fed a normal diet (ApoE^−/−^ ND), ApoE^−/−^ mice fed a high-cholesterol diet (ApoE^−/−^ HD), ApoE^−/−^ mice fed rosuvastatin calcium+a high-cholesterol diet (ApoE^−/−^ HD+R), and ApoE^−/−^ mice fed rosuvastatin calcium+sarpogrelate+a high-cholesterol diet (ApoE^−/−^ HD+R+S), all for 8 weeks (*n* = 6 in each group).

In an initial histochemical survey, hematoxylin and eosin- (H&E-) stained sections of ApoE^−/−^ mice fed a normal diet (ApoE^−/−^ ND) showed that all the components of the renal cortex appeared grossly normal, including renal corpuscles, convoluted tubules with Henle's loop, cortical collecting ducts, and other blood vessels, exhibiting the normal phenotypes in the glomerular capillary, Bowman's space, macula densa, distal convoluted tubules, and proximal convoluted tubules composed of cuboidal cells with the apical brush border, acidophilic cytoplasm, and rounded nuclei under an upright microscope (Figure 1(a)). Accordingly, representative images of the renal cortex of ApoE^−/−^ mice fed a high-cholesterol diet (ApoE^−/−^ HD) revealed glomerular endothelial proliferation and mesangial expansion. Also, tubular damage and extracellular matrix accumulation, namely, a marked increase in the macula densa, scattered and apoptotic loss of the apical brush border with vacuolated basophilic cytoplasm and heavily stained nuclei, and expanded Bowman's space (Figure 1(b)). On the contrary, representative images of the renal cortex of ApoE^−/−^ mice fed rosuvastatin calcium+a high-cholesterol diet (ApoE^−/−^ high-fat diet (HD)+R) showed relatively less dramatic changes in the aforementioned structural components (Figure 1(c)). In particular, a combinational use of sarpogrelate and rosuvastatin calcium in ApoE^−/−^ mice fed rosuvastatin calcium+sarpogrelate+a high-cholesterol diet (ApoE^−/−^ HD+R+S) obviously ameliorated the pathological changes compared with that in mice from the ApoE^−/−^ HD or ApoE^−/−^ HD+R group (Figure 1(d)). In brief, these results indicated that rosuvastatin and sarpogrelate indeed ameliorated the dramatic pathological changes caused by HD in ApoE-deficient mice.

### 3.2. Rosuvastatin/Sarpogrelate-Mediated Reduced Expression of Proinflammatory Genes Was Involved in the Amelioration of Pathological Features of the Renal Cortex in ApoE-Deficient Mice Subjected to a High-Fat Diet

**Figure 2:**
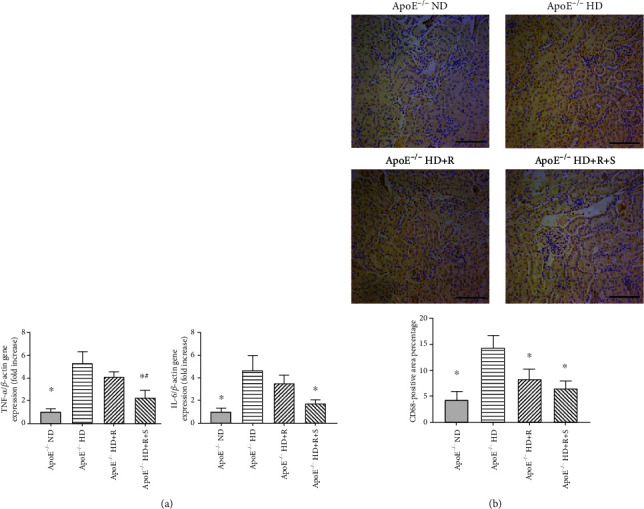
Detection of changes in expression levels of proinflammatory genes and pathological heterogeneity under each condition. (a) Relative mRNA expression of TNF-*α* and IL-6 was evaluated by qPCR in the kidney tissues of mice following an 8-week treatment under each condition (*n* = 6 in each group). (b) Representative images and quantification of immunohistochemistry showing the CD68 expression level in kidney tissues of mice following an 8-week treatment under each condition. Scale bar, 200 mm (*n* = 3–4 in each group). Data were expressed as mean ± SEM; ^∗^*P* < 0.05 vs. ApoE^−/−^ HD, ^#^*P* < 0.05 vs. ApoE^−/−^ HD+R.

The kidney tissue of mice was tested following 8-week treatment under each condition by evaluating the expression levels of proinflammatory genes using real-time quantitative polymerase chain reaction (qPCR) to determine whether the amelioration of dramatic pathological changes in mice from the ApoE^−/−^ HD group would affect the expression levels of proinflammatory genes in glomerular disease (Table 1). Further, a significant increase in the expression levels of TNF-*α* and IL-6 mRNA was found in mice from the ApoE^−/−^ HD or ApoE^−/−^ HD+R group, consistent with their predominant proinflammatory influences on the renal cortex, compared with that from the ApoE^−/−^ ND group (^∗^*P* < 0.05 vs. ApoE^−/−^ HD; ^#^*P* < 0.05 vs. ApoE^−/−^ HD+R). However, they were almost unaffected in mice from the ApoE^−/−^ HD+R+S group (Figure 2(a)). Likewise, CD68, which is highly expressed by proinflammatory cell types including monocyte lineage and macrophages, was also tested using immunohistochemistry on sections containing renal cortices. The mice from the ApoE^−/−^ HD group exhibited increased expression levels of CD68, which was almost reversed by rosuvastatin and/or sarpogrelate treatment in mice from the ApoE^−/−^ HD+R or ApoE^−/−^ HD+R+S group (^∗^*P* < 0.05 vs. ApoE^−/−^ HD) (Figure 2(b)). Therefore, these results suggested that rosuvastatin and sarpogrelate were indeed involved in reducing the CD68-positive monocyte/macrophage-mediated proinflammatory activity induced by HD in ApoE-deficient mice.

### 3.3. Rosuvastatin/Sarpogrelate-Mediated Modulation of Cholesterol Homeostasis Contributed to the Amelioration of Pathological Features of the Renal Cortex in ApoE-Deficient Mice Subjected to a High-Fat Diet

**Figure 3:**
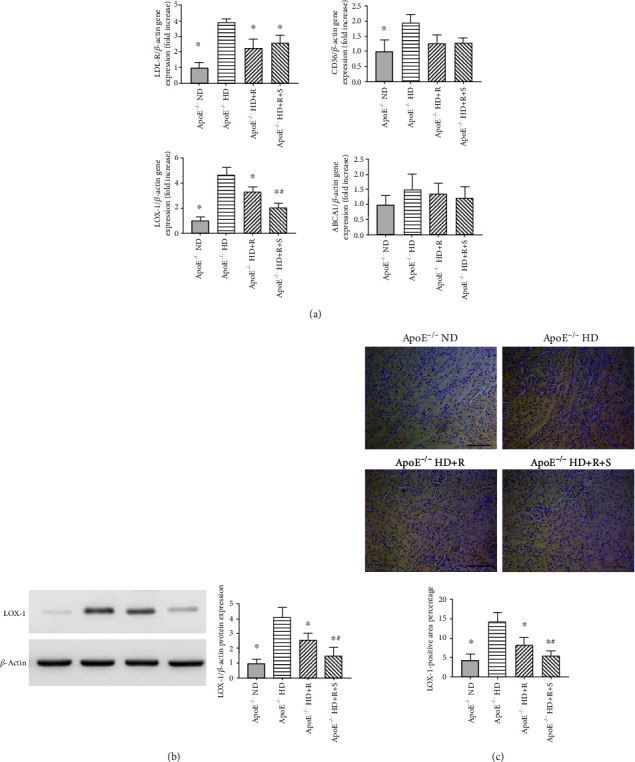
Detection of alterations of lipid homeostasis and pathological heterogeneity under each condition. (a) Relative mRNA expression of LDL-R, CD36, LOX-1, and ABCA1 was evaluated by qPCR in the kidney tissues of mice following an 8-week treatment under each condition (*n* = 6 in each group). (b) Representative images and quantifications of Western blot analysis showing the LOX-1 expression level in kidney tissues of mice following an 8-week treatment under each condition. Scale bar, 200 mm (*n* = 3–4 in each group). (c) Representative images and quantification of immunohistochemistry showing the LOX-1 expression level in kidney tissues of mice following an 8-week treatment under each condition. Scale bar, 200 mm (*n* = 3–4 in each group). Data were expressed as mean ± SEM; ^∗^*P* < 0.05 vs. ApoE^−/−^ HD, ^#^*P* < 0.05 vs. ApoE^−/−^ HD+R.

Considering a significant reduction in the expression levels of proinflammatory genes in the renal cortex treated with rosuvastatin/sarpogrelate, this study next explored their modulatory effects on lipid homeostasis. It was speculated that rosuvastatin/sarpogrelate treatment would affect the regulation of cholesterol efflux, in particular of LDL cholesterol, and its complication in ApoE-deficient mice with HD-induced chronic renal failure. Treatment of HD in ApoE-deficient mice resulted in an apparent increase in the expression levels of LDL-R and LOX-1, which were almost reversed by rosuvastatin and/or sarpogrelate treatment (^∗^*P* < 0.05 vs. ApoE^−/−^ HD; ^#^*P* < 0.05 vs. ApoE^−/−^ HD+R) (Figure 3(a)). Interestingly, the study also found no significant changes in the expression levels of CD36 and ABCA1, indicating that neither the regulation of ABCA1-mediated efflux of cholesterol nor the CD36-mediated ligand binding and internalization was affected (Figure 3(a)). Accordingly, the kidney weight, total body weight, and levels of total cholesterol (TC), triglyceride (TG), and low-density lipoprotein (LDL) cholesterol (LDL-c) were also measured. The results revealed a similar trend in some of these features compared with other methods used, although the body weight was not affected under all the conditions and LDL-c was even more increased in mice from the ApoE^−/−^ HD+R group compared with mice from the ApoE^−/−^ HD group (Table 2). Moreover, the expression of LOX-1 induced by LDL accumulation on endothelial cells was tested by Western blot analysis and immunohistochemistry. Likewise, rosuvastatin and/or sarpogrelate treatment did reverse the increased expression level of LOX-1, which was observed in mice from the ApoE^−/−^ HD group, indicating excessive proinflammatory release, endothelial dysfunction, and other active immune responses in these mice (^∗^*P* < 0.05 vs. ApoE^−/−^ HD; ^#^*P* < 0.05 vs. ApoE^−/−^ HD+R) (Figures 3(b) and 3(c)). Taken together, these results suggested that rosuvastatin and sarpogrelate indeed contributed to the amelioration of pathological features of the renal cortex through restoring the cholesterol homeostasis of endothelial cells in ApoE-deficient mice subjected to HD.

## 4. Discussion

Hyperlipidemia, as one of the major metabolic disorders of comorbidities in patients with CKD, often inevitably leads to atherosclerosis and subsequent platelet-stimulated blood coagulation in progressive renal failure [27, 28]. In recent years, rosuvastatin, as an antiatherogenic drug, and sarpogrelate, also known as an antiplatelet agent, have been commonly used in clinical trials [21]. This study is aimed at investigating whether the synergistic action of sarpogrelate and rosuvastatin could potentially accelerate and ameliorate CKD progression in patients with hyperlipidemia and CKD. In the result, a combination of sarpogrelate and/or rosuvastatin was used on HD-induced CKD mice for 8 weeks. LDL-R- or SR- or LOX-1-positive monocyte/macrophage-mediated enhanced immune response, such as the upregulation of TNF-*α* and IL-6, was indeed ameliorated by sarpogrelate and/or rosuvastatin treatment. The improvement is more effective due to the concurrence of sarpogrelate and rosuvastatin. The treatment also partially reversed the tremendous alterations in glomerular damages, extracellular matrix accumulation, and others, detected using real-time qPCR, Western blot analysis, and immunohistochemistry. It showed that rosuvastatin was an important treatment for reduced expression of proinflammatory genes and modulation of cholesterol homeostasis contributed to the amelioration of pathological features of the renal cortex in ApoE-deficient mice subjected to a high-fat diet. The synergistic action of the two treatments potentially accelerates and ameliorates the progression.

A research showed that combinatorial treatment with sarpogrelate and rosuvastatin was relatively more effective in ameliorating histopathological changes when compared to the single-drug treatment, which could effectively ameliorate HFD/STZ-induced CKD progression by improving histopathological changes in the glomerulus and tubules and attenuating interstitial fibrosis, albuminuria, and urinary cystatin C excretion. Combinatorial treatment inhibited profibrotic PAI-1 expression in HFD/STZ mouse kidney and mesangial cells [21]. Another recent report showed the beneficial effects of the combinational use of sarpogrelate and rosuvastatin in HD-induced nephropathic mice and streptozotocin, providing numerous insights into the potential mechanisms and curative effects [21]. However, the use of the alkylating antineoplastic agent streptozocin, which is toxic to insulin-producing beta cells in mammals, potentially caused fundamental changes owing to the involvement of various causative factors in the pathogenesis of CKD, considering diabetic nephropathy as the leading cause of CKD and the essential role of insulin in diabetic nephropathy [29–31].

High-cholesterol diet-induced CKD increased LDL production and release, reflected by an increase in TC and LDL-c. This was followed by overwhelming inflammatory reactions, including the production of proinflammatory cytokines (TNF-*α* and IL-6) and LOX-1/CD68-mediated recirculation through the association of endosomes and lysosomes with the plasma membrane. This allowed the activation of reactive inflammatory cells such as macrophages to selectively interact with related cells or substrates. Interestingly, CD36 is not as sensitive as other SRs tested in this study, making it a bit peculiar compared with LOX-1/CD68 among other SRs. However, it is involved in a number of related processes, such as binding, internalization, and oxidization of LDLs, phospholipids, and long-chain fatty acids. These results indicated that other mechanisms were activated simultaneously to neutralize the increasing effect on CD36 during CKD progression under certain conditions. Therefore, further investigations should be conducted to thoroughly understand the precise role of CD36 in high-cholesterol diet-induced CKD in ApoE-deficient mice. Efforts are needed in the future to develop effective and efficient treatments for CKD progression. An accurate and noninvasive treatment, such as a combinational use of sarpogrelate and/or rosuvastatin, is an invaluable way to achieve a better prognosis in patients with certain metabolic comorbidities across various CKD-affected populations.

In addition, the limitation of this research is its small group size, which could affect the result of the experiment. We will attempt to avoid it in our follow-up experiment. Moreover, the study only focused on comparison with rosuvastatin alone as we aimed to investigate the synergistic action of the two drugs and identify the corresponding mechanisms but omitted comparison with sarpogrelate. In our future research, it will be necessary to be perfect.

## 5. Conclusions

Mice with CKD induced by a high-fat diet were treated with sarpogrelate and/or rosuvastatin for 8 weeks. Sarpogrelate and/or rosuvastatin treatment alleviates the histopathological changes of CKD in the kidney of the mice; the synergistic action of the two drugs accelerates the changes. Sarpogrelate and/or rosuvastatin treatment attenuates the upregulation of tumor necrosis factor-*α* and interleukin-6 induced by CKD, as well as the LDL-R/CD68/LOX-1-positive monocyte/macrophage-mediated enhanced proinflammatory activation. Actually, combination of sarpogrelate and rosuvastatin was more effective. The findings suggest that rosuvastatin treatment could potentially ameliorate CKD progression and the synergistic action of the two drugs will enhance and ameliorate CKD progression which is a very important role in patients with hyperlipidemia and CKD, but clinical trials are necessary to answer this question.

## Figures and Tables

**Table 1:** Oligonucleotide primer sequences.

LOX-1, lectin-like oxidized low-density lipoprotein receptor-1:
F: 5′-CAAAGTCTCCCAACCAACCTGCAA-3′
R: 5′-ACATCCTGTCTTTCATGCGGCAAC-3′
LDL-R, the low-density lipoprotein receptor:
F: 5′-TTGGGTTGATTCCAAACTCCAT-3′
R: 5′-CCGATTGCCCCCATTGA-3′
CD36, cluster of differentiation 36, also known as scavenger receptor class B member 3 (SCARB3):
F: 5′-CCTTAAAGGAATCCCCGTGT-3′
R: 5′-TGCATTTGCCAATGTCTAGC-3′
ABCA1, ATP-binding cassette transporter A1:
F: 5′-AGCCAGAAGGGAGTGTCAGA-3′
R: 5′-CATGCCATCTGGGTAAACCT-3′
TNF-*α*, tumor necrosis factor-*α*:
F: 5′-TCTCATGCACCACCATCAAGGACT-3′
R: 5′-ACCACTCTCCCTTTGCAGAACTCA-3′
IL-6, interleukin-6:
F: 5′-TACCAGTTGCCTTCTTGGGACTGA-3′
R: 5′-TAAGCCTCCGACTTGTGAAGTGGT-3′
*β*-Actin:
F: 5′-CGATGCCCTGAGGGTCTTT-3′
R: 5′-TGGATGCCACAGGATTCCAT-3′

**Table 2:** Metabolic data.

	ApoE^−/−^ ND	ApoE^−/−^ HD	ApoE^−/−^ HD+R	ApoE^−/−^ HD+R+S
Body weight (g)	30 ± 2.65	28.4 ± 2.61	29.5 ± 2.35	29.5 ± 1.52
Kidney/body weight ratio (mg g^−1^)	6.45 ± 0.1	7.22 ± 0.86	6.98 ± 0.87	7.04 ± 1.13
TC (mg dL^−1^)	9.7 ± 0.68^∗^^#^	102.33 ± 8.17	78.28 ± 21.25	40.9 ± 184^∗^^#^
TG (mg dL^−1^)	2.87 ± 0.85	3.13 ± 0.81	2.08 ± 0.39	1.4 ± 0.28
LDL-c (mg dL^−1^)	3.95 ± 0.32^∗^^#^	17.9 ± 0.07	14.7 ± 2.12	10.33 ± 0.18^∗^

The mice were randomly divided into four groups: ApoE^−/−^ mice fed a normal diet (ApoE^−/−^ ND), ApoE^−/−^ mice fed a high-cholesterol diet (ApoE^−/−^ HD), ApoE^−/−^ mice fed rosuvastatin calcium+a high-cholesterol diet (ApoE^−/−^ HD+R), and ApoE^−/−^ mice fed rosuvastatin calcium+sarpogrelate+a high-cholesterol diet (ApoE^−/−^ HD+R+S). After the sacrifice following an 8-week treatment, the kidney weight and the total body weight of the mice from each group were measured and recorded. Blood samples were obtained from their inferior vena cava and collected. The levels of total cholesterol (TC), triglycerides (TG), and low-density lipoprotein cholesterol (LDL-c) were measured with an autoanalyzer system. Data were expressed as mean ± SEM (*n* = 5–6 in each group). ^∗^*P* < 0.05 vs. ApoE^−/−^ HD, ^#^*P* < 0.05 vs. ApoE^−/−^ HD+R.

## Data Availability

The data used to support the findings of this study are included within the article.
